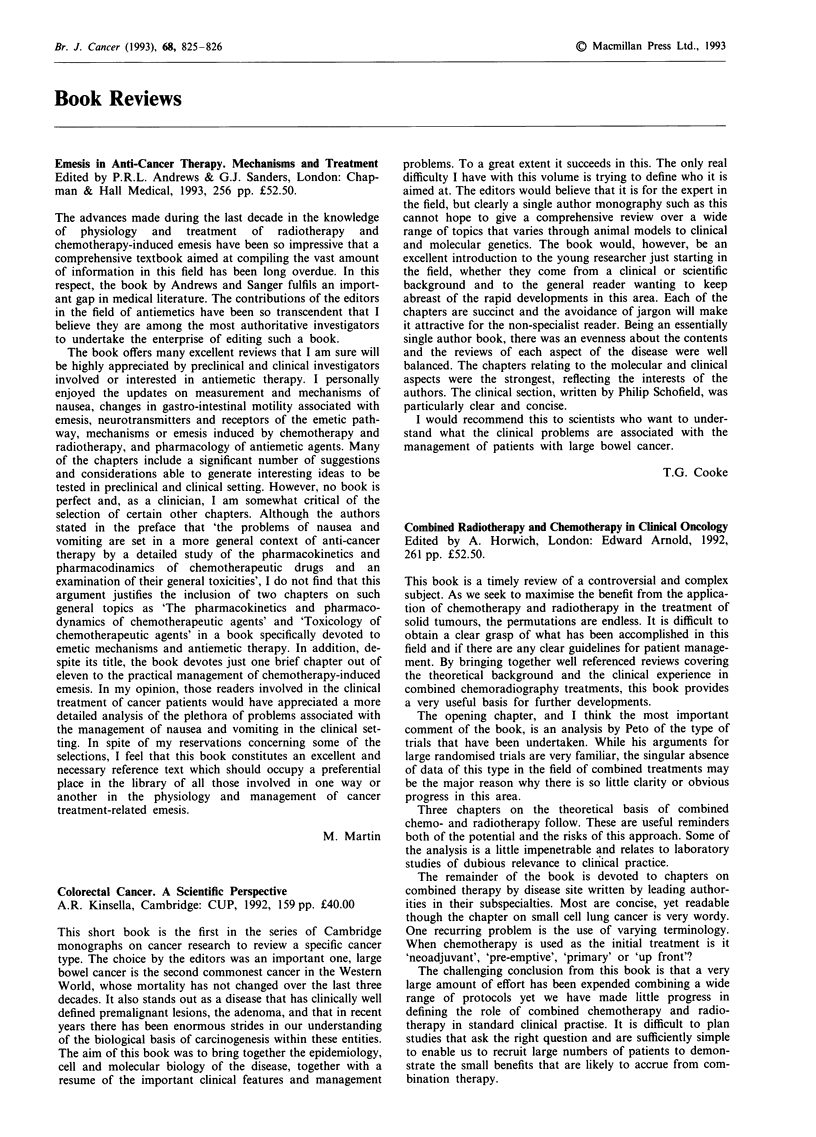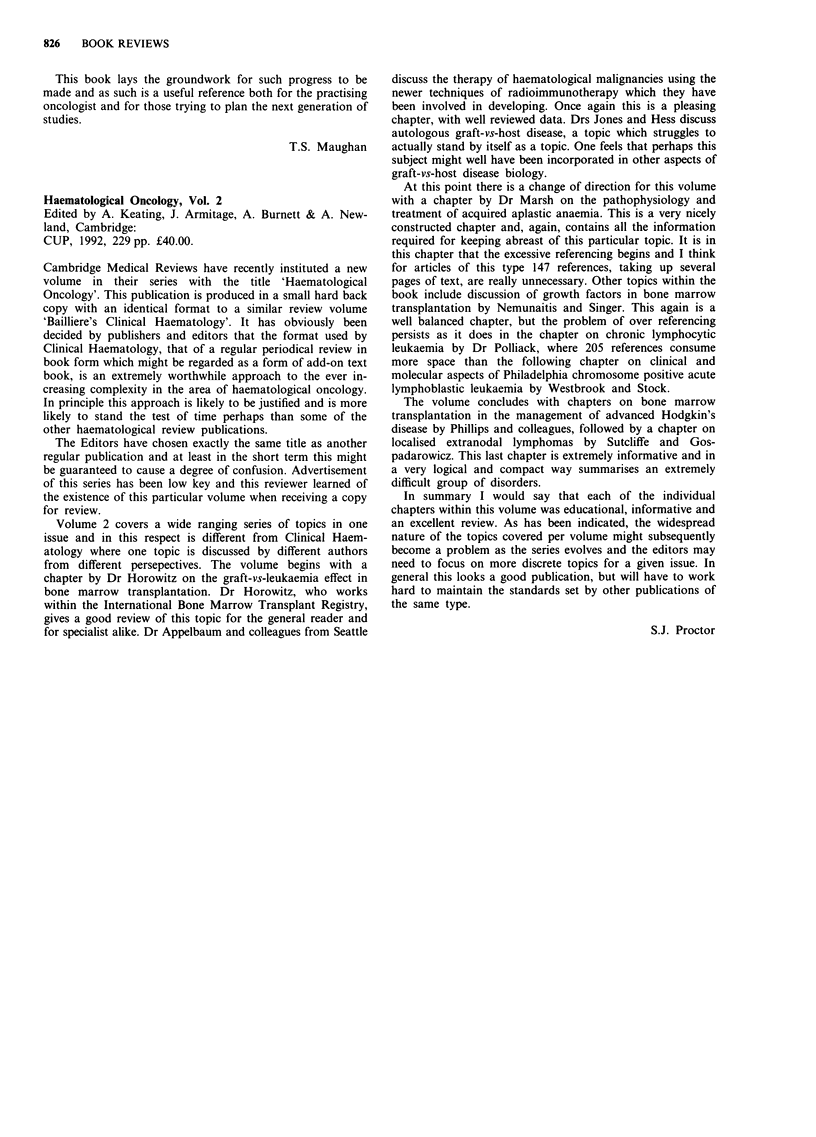# Combined Radiotherapy and Chemotherapy in Clinical Oncology

**Published:** 1993-10

**Authors:** T.S. Maughan


**Combined Radiotherapy and Chemotherapy in Clinical Oncology**
Edited by A. Horwich, London: Edward Arnold, 1992, 261 pp. £52.50.

This book is a timely review of a controversial and complex subject. As we seek to maximise the benefit from the application of chemotherapy and radiotherapy in the treatment of solid tumours, the permutations are endless. It is difficult to obtain a clear grasp of what has been accomplished in this field and if there are any clear guidelines for patient management. By bringing together well referenced reviews covering the theoretical background and the clinical experience in combined chemoradiography treatments, this book provides a very useful basis for further developments.

The opening chapter, and I think the most important comment of the book, is an analysis by Peto of the type of trials that have been undertaken. While his arguments for large randomised trials are very familiar, the singular absence of data of this type in the field of combined treatments may be the major reason why there is so little clarity or obvious progress in this area.

Three chapters on the theoretical basis of combined chemo- and radiotherapy follow. These are useful reminders both of the potential and the risks of this approach. Some of the analysis is a little impenetrable and relates to laboratory studies of dubious relevance to clinical practice.

The remainder of the book is devoted to chapters on combined therapy by disease site written by leading authorities in their subspecialties. Most are concise, yet readable though the chapter on small cell lung cancer is very wordy. One recurring problem is the use of varying terminology. When chemotherapy is used as the initial treatment is it 'neoadjuvant', 'pre-emptive', 'primary' or 'up front'?

The challenging conclusion from this book is that a very large amount of effort has been expended combining a wide range of protocols yet we have made little progress in defining the role of combined chemotherapy and radiotherapy in standard clinical practise. It is difficult to plan studies that ask the right question and are sufficiently simple to enable us to recruit large numbers of patients to demonstrate the small benefits that are likely to accrue from combination therapy.

This book lays the groundwork for such progress to be made and as such is a useful reference both for the practising oncologist and for those trying to plan the next generation of studies.

T.S. Maughan